# A Simple and Safe Technique for Manipulation of Retrosternal Dissection in the Nuss Procedure

**Published:** 2014-02-05

**Authors:** Masahiko Noguchi, Shoji Kondoh, Kenya Fujita

**Affiliations:** ^a^Department of Plastic and Reconstructive Surgery, Nagano Children's Hospital, Toyoshina, Azumino, Japan; ^b^Department of Plastic and Reconstructive Surgery, Shinshu University School of Medicine, Matsumoto, Japan; ^c^Department of Plastic Reconstructive Surgery, Ina Central Hospital, Ina, Japan

**Keywords:** Nuss procedure, pectus excavatum, prevent life-threatening complication, safe dissection, the layer of the dissection level

## Abstract

**Objective:** The Nuss procedure has become the first choice for repairing the pectus excavatum because of the advantages of the technique including minimal invasiveness and short operative duration. Although this technique appears simple and easy, life-threatening complications during dissection such as intraoperative cardiac perforation have been reported. We developed a new approach for safer dissection of retrosternal space. **Methods:** We use a dissector that is commonly used for laparoscopic operation, instead of the Nuss introducer. The dissector goes through the same skin incision where the Nuss bar will be inserted. The major difference is the position of dissector insertion, which is set up more dorsally than usual, and the use of a laparoscopic dissector instead of the Nuss introducer. In this new approach, the direction of dissection is from dorsal toward the anterior thoracic wall, which allows us to visually follow the tip of the dissector throughout the surgery. Moreover using the dissector that has better manipulation capability enables us the fine dissection and also is able to precisely determine both the layer and the area of the dissection level. **Results:** We have treated more than 150 patients using this technique without any complications since 2008. In all the cases, safer dissection of the retrosternal space was performed with good results. **Conclusion:** We believe every surgeon can easily apply this procedure to patients with pectus excavatum, and this procedure can reduce the stress during the dissection.

Although the Nuss procedure is a widely used minimally invasive method for repair of pectus excavatum, some catastrophic complications have been reported, including life-threatening intraoperative cardiac perforation.^1^^-^6 Thoracoscopic surgery was proposed for safer dissection between the sternum and the heart.^7^^-^9 Although this method has advantages, it still does not fulfill our safety requirements. Many other supportive methods have been reported to obtain a better visual field.^10^^,^^11^ In our institute, we experienced 2 cases of pericardiac perforation and 1 case of injury of the internal thoracic artery during retrosternal dissection.

Here, we introduce a new technique for safer retrosternal dissection, which involves a slight shift of the bar insertion point and the use of a laparoscopic dissector instead of the Nuss introducer. This seemingly simple modification makes a great difference in visibility of the field of operation and manipulation of surgical tools, leading the Nuss procedure to much safer technique. We believe this procedure can reduce the stress during dissection in patients with pectus excavatum.

## CASE REPORT

**Figure 1:**
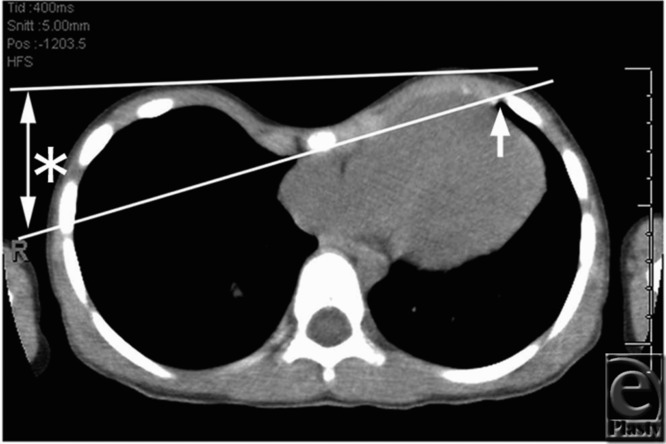
A 9-year-old boy with symmetric chest wall deformity. The insertion point for the dissector was determined by CT examination. The insertion point of the Nuss bar on the left thoracic wall (↑). The depth between the body surface and the insertion point (*) was 7.5 cm.

**Figure 2:**
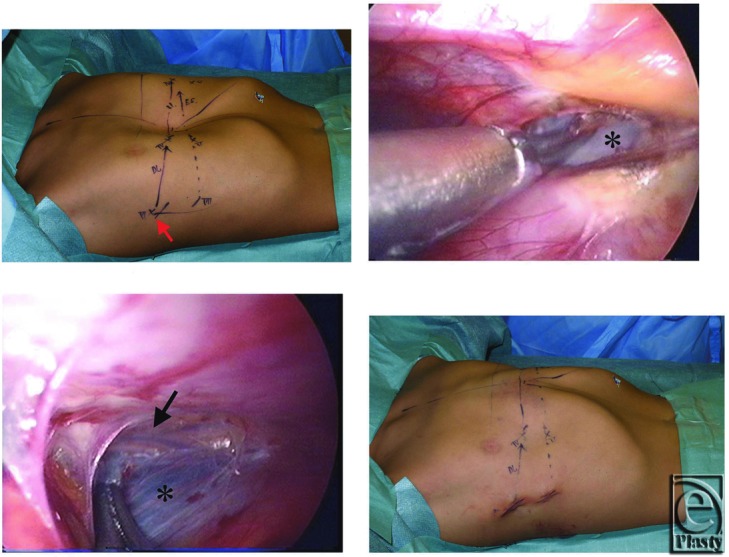
Intraoperative view of the same patient shown in Fig 1. (*a*) Intraoperative design. The dissector passed through from the same skin incision where the Nuss bar was to be inserted. (*b*) The right side of the mediastinum. Dissection of the mediastinum. The layer of the dissection was just above the pericardium (*). (*c*) The left pleura (*) and internal thoracic vessels (↑). Sufficient tissues were left in place to protect the internal thoracic vessels from damage. (*d*) Postoperative view.

The patient was a 9-year-old boy with a symmetric chest wall deformity. As the first step, the insertion point of the dissector on the right thoracic wall was determined using a CT image taken in the axial plane where the Nuss bar was to be inserted. On this image, a line was drawn from the insertion point of the Nuss bar on the left thoracic wall (Fig 1, *white arrow*) along the retrosternal plane (Fig 1, *red arrow*). The point at which this line extended to meet the lateral portion of the right thoracic wall was the insertion point of the dissector. The depth between the body surface and the insertion point was 7.5 cm in this case (Fig 1*). From this evaluation, the dissector was inserted from the sixth intercostal space near the mid-axillary line (Fig 2a ↑) and therefore skin incision was set up dorsally from the usual position. In dissection of the retrosternal space, we dissected just above the pericardia not just beneath the sternum to preserve sufficient tissues around both cardiac and internal thoracic vessels to protect them from damage (Figs 2b and 2c). The dissection manipulation was smooth and the bar was placed at the fourth intercostal space. A good result was obtained (Fig 2d).

## DISCUSSION

In a study of 167 children, Castellani reported that 4.2% of patients sustained major complications, including intraoperative heart perforation.^5^ In 2009, Bouchard reviewed 4 cases of cardiac injury during the Nuss procedure. Since then, the role of thoracoscopy has been well established. However, cases of cardiac perforation persist despite its use.^1^ He suggested that different approaches could also be used to guide passage of the pectus dissector in the anterior mediastinum. In our institute, we had experienced 2 cases of pericardiac perforation and 1 case of injury of the internal thoracic artery during retrosternal dissection with ordinary introducer under the thoracoscopy before the introduction of our new technique.

**Figure 3:**
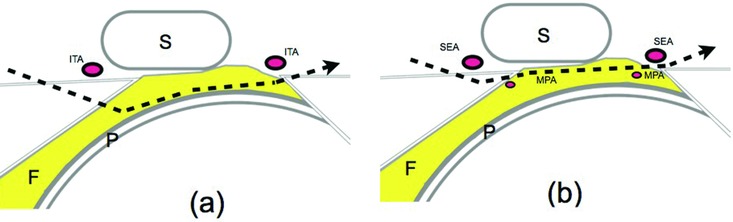
Transverse sections showing the paths of a dissector in our new technique. A dissector goes through the route (*a*) when the bar is inserted rostrally to the bifurcation of MPA, while a dissector takes the route (*b*) when the bar is to be placed caudally to the bifurcation of MPA. F indicates fatty falls of pleura; ITA, internal thoracic artery; MPA, musculophrenic artery; P, pericardium; S, caudal portion of the sternum; SEA, superior epigastric artery.

Most commonly, for a better result, the bar is placed under the most concave portion of the sternum in Nuss procedure. However, in this area, a fat pad, so-called the fatty falls of pleura, exists on the pericardium. Major arteries run in and around the fat pad. The internal thoracic artery runs anterior to the fat pad. The musculophrenic artery that is the branch from the internal thoracic artery runs through in the fat pad (Fig 3). Therefore, precise dissection is required to prevent these vasculatures from injuries. Moreover, the pericardium is tightly attached to the diaphragm at this portion, blunt dissection blindly would be more challenging and risky compared to the other part of the anterior mediastinum.

In the Nuss procedure, the introducer is regularly used for the dissection. The introducer is inserted from the highest point of the anterior chest wall and the dissection is carried out concurrent with elevation of the concave thorax. These conditions make the precise manipulations difficult and lead to inadequate dissection. Our new approach overcomes these problems. In comparison to the traditional method, our procedure makes insertions at more posterior points. As a result, we can keep the tip of the dissector in view until it reaches the contralateral insertion point of the bar, which in turn makes it possible to guide the dissector in a safer direction. In addition to the increased visibility, the manipulation capability of the dissector is also improved in our method. Using this approach, we are able to precisely determine both the layer and the area of the dissection level. In the case that the bar is placed cephalad to the bifurcation of the musculophrenic artery, we prefer to dissect just above the pericardia, which is considered beneficial for reducing intraoperative injury of both the cardiac and internal thoracic vessels (Fig 3a). On the contrary, when the bar is placed caudally to the bifurcation of the musculophrenic artery, we dissect between the superior epigastric artery and the musculophrenic artery (Fig 3b). In the case of extremely severe asymmetry, the insertion point of the dissector becomes more posterior and difficult to manipulate. In such cases, we use a vacuum chest wall lifter at the same time to ensure definite and safe dissection. It is noteworthy that we have not had any complications over 150 patients since we started this new technique.

## References

[1] Bouchard S, Hong AR, Gilchrist BF, Kuenzler KA (2009). Catastrophic cardiac injuries encountered during the minimally invasive repair of pectus excavatum. Semin Pediatr Surg.

[2] Hebra A, Swoveland B, Egbert M (2000). Outcome analysis of minimally invasive repair of pectus excavatum: review of 251 cases. J Pediatr Surg.

[3] Moss RL, Alabanese CT, Reynolds M (2001). Major complications after minimally invasive repair of pectus excavatum: case reports. J Pediatr Surg.

[4] Belcher E, Arora S, Samancilar O, Goldstraw P (2008). Reducing cardiac injury during minimally invasive repair of pectus excavatum. Eur J Cardiothorac Surg.

[5] Castellani C, Schalamon J, Saxena AK, Hoellwarth ME (2008). Early complications of the Nuss procedure for pectus excavatum: a prospective study. Pediatr Surg Int.

[6] Gips H, Zaitsev K, Hiss J (2008). Cardiac perforation by a pectus bar after surgical correction of pectus excavatum: case report and review of the literature. Pediatr Surg Int.

[7] Nuss D (2008). Minimally invasive surgical repair of pectus excavatum. Semin Pediatr Surg.

[8] Saxena AK, Castellani C, Hollwarth ME (2007). Surgical aspects of thoracoscopy and efficacy of right thoracoscopy in minimally invasive repair of pectus excavatum. J Thorac Cardiovasc Surg.

[9] Croitoru DP, Kelly RE, Goretsky ML, Lawson ML, Swoveland B, Nuss D (2002). Experience and modification update for the minimally invasive Nuss technique for pectus excavatum repair in 303 patients. J Pediatr Surg.

[10] Hendrickson RJ, Bensard DD, Janik JS, Partrick DA (2005). Efficacy of left thoracoscopy and blunt mediastinal dissection during the Nuss procedure for pectus excavatum. J Pediatr Surg.

[11] Cheng YL, Lee SC, Huang TW, Wu CT (2008). Efficacy and safety of modified bilateral thoracoscopy-assisted Nuss procedure in adult patients with pectus excavatum. Eur J Cardiothorac Surg.

